# No Plasmatic Proteomic Signature at Clinical Disease Onset Associated With 11 Year Clinical, Cognitive and MRI Outcomes in Relapsing-Remitting Multiple Sclerosis Patients

**DOI:** 10.3389/fnmol.2018.00371

**Published:** 2018-10-31

**Authors:** Claire Bridel, Anand J. C. Eijlers, Wessel N. van Wieringen, Marleen Koel-Simmelink, Cyra E. Leurs, Menno M. Schoonheim, Joep Killestein, Charlotte E. Teunissen

**Affiliations:** ^1^Neurochemistry Laboratory, Department of Clinical Chemistry, Amsterdam Neuroscience, VU University Medical Center, Amsterdam, Netherlands; ^2^Department of Anatomy and Neurosciences, MS Center Amsterdam, Amsterdam Neuroscience, VU University Medical Center, Amsterdam, Netherlands; ^3^Department of Epidemiology and Biostatistics, VU University Medical Center, Amsterdam, The Netherlands and Department of Mathematics, VU University, Amsterdam, Netherlands; ^4^Department of Neurology, MS Center Amsterdam, Amsterdam Neuroscience, VU University Medical Center, Amsterdam, Netherlands

**Keywords:** multiple sclerosis, prognosis, proteomics, MRI, cognition

## Abstract

**Background:** The clinical course of relapsing-remitting multiple sclerosis (RRMS) is highly heterogeneous and prognostic biomarkers at time of diagnosis are lacking.

**Objective:** We investigated the predictive value of the plasma proteome at time of diagnosis in RRMS patients.

**Methods:** The plasma proteome was interrogated using a novel aptamer-based proteomics platform, which allows to measure the levels of a predefined set of 1310 proteins.

**Results:** In 67 clinically and radiologically well characterized RRMS patients, we found no association between the plasma proteome at diagnosis and clinical, cognitive or MRI outcomes after 11 years.

**Conclusions:** Proteomics studies on cerebrospinal fluid may be better suited to identify prognostic biomarkers in early RRMS.

## Introduction

The clinical course of relapsing-remitting multiple sclerosis (RRMS) is highly heterogeneous and prognostic biomarkers at time of diagnosis are lacking. Early identification of patients at risk of an unfavorable mid- to long-term outcome is critical, as this knowledge may help select the most appropriate therapeutic strategy and provide adequate counseling. Converging evidence support the hypothesis that auto reactive T-cells primed outside the central nervous system (CNS) are key to the pathogenesis of MS (McFarland and Martin, 2007; Legroux and Arbour, 2016). We hypothesized that the blood proteome is modified by early pathological processes, and is a source of prognostic biomarkers at time of diagnosis. Several studies have used (immuno)proteomics tools to analyze the blood proteome in MS, with the aim of identifying diagnostic biomarkers and dysregulated pathways which may shed light on the pathogenenic intricacies of MS (Almeras et al., 2004; Avasarala et al., 2005; Rithidech et al., 2009; Teunissen et al., 2011; Liu et al., 2012; Byström et al., 2014; Colomba et al., 2014; Jiang et al., 2014; Yin et al., 2014). However, none of these studies have related the proteomic changes to long term outcomes. Here, we investigated whether a protein expression signature was associated with clinical, cognitive or MRI outcomes, 11 years after diagnosis in a cohort of 67 well characterized RRMS patients. Biobanked plasma was interrogated using a novel aptamer-based multiplexed proteomic platform, which allows to measure levels of 1310 predetermined proteins including cell signaling molecules, receptors and proteins involved in neuronal repair (Gold et al., 2010).

## Methods

### Patients

This study was performed according to the ethical principles of the Declaration of Helsinki and was approved by the local ethics committee (VUmc, Amsterdam, Netherlands). All patients signed informed consent for the use of plasma and clinical information. Patients were recruited on a voluntary basis at the MS center of the VU University Medical Center, Amsterdam, Netherlands. At year 11, patients fulfilled the 2001 revisions of the McDonald criteria for RRMS (McDonald et al., 2001). During follow-up, disease modifying therapies (DMTs) were introduced by the treating neurologist in 47.8% of patients. Of these, 68.7% received first line therapies only, while 33.1% subsequently received second line DMTs. No healthy controls were included in this study, as the aim was to explore the predictive value of the plasma proteome in RRMS patients.

### Plasma

Plasma was collected within 3 months of diagnosis, but at least 6 weeks from a relapse (referred to as baseline), and processed and stored according to international state-of-the-art guidelines on biobanking (Teunissen et al., 2009).

### Clinical and Cognitive Measures

Expanded disability status scores (EDSS) were determined by certified clinicians at baseline and year 11 for the whole cohort (*n* = 67), and ΔEDSS was calculated. Cognitive performance in seven different cognitive domains was determined at year 11 for 49 patients. Cognitive test scores were transformed into domain-specific Z-scores, based on a matched healthy control group (*n* = 29), and a global cognitive score was calculated as published (Eijlers et al., 2017).

### MRI Measures

MRI was acquired at year 11 for 46 patients using a General Electric 3 Tesla MRI system with an 8-channel head coil. Normalized whole brain volume and normalized thalamic volume were measured on 3-dimensional T1-weighted images using SIENAX and FIRST [both part of FSL (Smith et al., 2004)] and transformed into Z-scores, based on volumes in a matched healthy control group (*n* = 59), as done previously (Eijlers et al., 2018). White matter lesion volume was calculated on fluid-attenuated inversion recovery images using an automated segmentation technique (Steenwijk et al., 2013).

### Plasma Proteomics

The proteome of each plasma sample was assessed using the SOMAscan (SomaLogic, Boulder, CO, United States), a multiplexed proteomic platform using protein-capture SOMAmers (Slow Off-rate Modified Aptamers) to measure the levels of a predefined set of 1310 proteins (Gold et al., 2010). Aptamers are single stranded DNA molecules that are modified to mimic protein side chains, which allow selective binding to protein targets of interest. Moreover, theses DNA aptamers are able to pair with DNA probes, allowing quantification using a DNA microarray. Therefore, the measurement output is in relative fluorescence units (RFU). The reported intra and inter-run coefficient of variation (CV%) in blood are <5% (Gold et al., 2010). Two levels of quality controls were performed; (1) to avoid relative signal variation between the samples at the microarray level, a set of aptamers with the lowest overall signal variation was used for normalization within plates, (2) the median RFU for a set of aptamer used for normalization was calculated from all the samples and a scaling factor for each individual sample was calculated to control for the technical variability.

### Outcome Measures

Four dichotomic outcome measures were defined: (i) EDSS outcome; EDSS progression was defined as a ΔEDSS ≥ 1; (ii) cognitive impairment; impaired cognition was defined as a cognitive score of Z < −2 in two or more cognitive domains compared to healthy controls; (iii) whole brain volume outcome; whole brain atrophy was defined as a normalized brain volume Z < −2 compared to healthy controls; (iv) thalamic volume outcome; thalamic atrophy was defined as normalized thalamic volume Z < −2 compared to healthy controls. A fifth, continuous outcome, consisted of white matter lesion volume at year 11.

### Statistical Analysis

Statistical analysis was performed is SPSS (version 20.0.0.0) and R (version 3.2.3). Proportions for categorical variables and mean values for continuous variables were compared using chi-square, student’s *t*-test and the Mann–Whitney test. Differentially expressed proteins were identified using the Limma-package in R, which implements a moderated *t*-test followed by the FDR (False Discovery Rate) multiplicity correction. Predictive power was assessed by the global test (Goeman et al., 2004) and penalized regression. A generalized linear regression model linking the outcome to the protein data was fitted in penalized -lasso, ridge and elastic net- fashion by means of the “glmnet” R-package. Performance of the thus built predictor was evaluated on left-out samples in the outer loop, with penalty parameter(s) determined through cross-validation in an inner loop.

## Results

**FIGURE 1:**
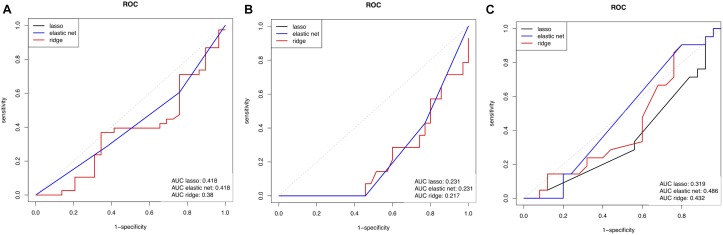
Receiver operating characteristic (ROC) curve showing the predictive performance of baseline proteome for **(A)** EDSS outcome measure, **(B)** global cognitive score measure, and **(C)** thalamic volume measure. Three curves are represented, acquired using 3 types of linear regression models to link the proteomic data to the outcomes. EDSS, expanded disability status score; AUC, area under the curve.

Demographic, clinical and MRI characteristics of the RRMS cohort are presented in Table 1. The cohort was dichotomized into outcome groups, according to the aforementioned four categorical outcome measures. ΔEDSS, cognitive scores, whole brain volume and thalamic volume were significantly different between outcome groups of the respective categorical outcome measure (Table 2). Mean age and the proportion of females did not differ between outcome groups for all four outcome measures (Table 2). The proportion of DMT-treated individuals did not differ between outcome groups for the EDSS, cognitive impairment and whole brain volume outcome measures (Table 2). For the thalamic volume outcome measure, the proportion of DMT-treated individuals was higher in those with thalamic atrophy at year 11 compared to those without (Table 2, *p* = 0.02). We could reliably measure 1310 proteins in all the plasma samples of the cohort, and the mean units per protein are presented in Supplementary Data Sheet S1. When we compared the global plasma protein expression profile at baseline between outcome groups for all four categorical outcome measures, we found no difference between individuals with or without EDSS progression (*p* = 0.651), with or without cognitive impairment (*p* = 0.802), with or without whole brain atrophy (*p* = 0.094), or with or without thalamic atrophy (*p* = 0.491). Moreover, we found no significant association between the proteomic profile and white matter lesion volume at year 11 (*p* = 0.111). Next, we assessed the classification performance of the proteomic features at baseline for three of the categorical outcomes, and found that no classifier could differentiate EDSS progression, cognitive impairment, or thalamic atrophy at 11-year (Figure 1). Because of the small number of individuals with whole brain atrophy at year 11 (*n* = 10), a classifier could not be built for this outcome measure.

**Table 1:** Clinical and radiological characteristics of the cohort of relapsing remitting multiple sclerosis patients (*n* = 67).

	**Mean age in years (SD)**	**Median EDSS (range)**	**Mean total brain volume Z-score compared to healthy controls (range)**	**Mean thalamic volume Z-score compared to healthy controls (range)**	**Mean global cognitive score Z-score compared to healthy controls (range)**	**% female**	**% DMT (% escalation)**
**Baseline**	32.7 (9.5)	2.0 (0 to 4.5)	NA	NA	NA	68.7	0
**Year 11**	43.7 (9.5)	3.0 (1.0–6.5)	−0.9 (−5.1 to 0.8)	−2.1 (−8.0 to −1.0)	−0.7 (−2.0 to 0.7)	68.7	47.8 (31.3)

**Table 2:** Number of individuals and clinical characteristics according to clinical cognitive or MRI outcome measures; EDSS scores were available at baseline and year 11 for all individuals (*n* = 67).

**Outcome measure i**		***N***	**Median EDSS at baseline (range)**	**Median EDSS at y 11 (range)**	**Mean age at baseline (SD) in years**	**% Female**	**% DMT (% escalation)**
EDSS	EDSS progression at y11	38	1.8 (0 to 4.0)	3.0 (1.0 to 6.5)	32.6 (10.3)	68.4	39.5 (10.5)
	No EDSS progression at y11	29	2.0 (1.0 to 4.5)	2.5 (1.0 to 4.0)	32.9 (8.5)	69.0	58.6 (20.7)
	*p*-value	.	*p* = 0.03	*p* < 0.001	*p* = 0.89	*p* = 0.96	*p* = 0.12 (*p* = 0.26)
**Outcome measure ii**		***N***	**Mean global cognitive score Z-score compared to healthy controls at baseline**	**Mean global cognitive score Z-score compared to healthy controls at y 11 (range)**	**Mean age at baseline (SD) in years**	**% Female**	**% DMT (% escalation)**
Global cognitive scale	Impaired cognition at y11	14	NA	−1.4 (−2.0 to −0.9)	34.1 (11.3)	57.1	50.0 (7.1)
	No impaired cognition at y11	35	NA	−0.5 (−1.4 to 0.7)	33.6 (7.5)	82.9	51.4 (17.1)
	*p*−value	.	NA	*p* < 0.001	*p* = 0.69	*p* = 0.21	*p* = 0.93 (*p* = 0.64)
**Outcome measure iii**		***N***	**Mean total brain volume Z-score compared to healthy controls at baseline**	**Mean total brain volume Z-score compared to healthy controls at y 11 (range)**	**Mean age at baseline (SD) in years**	**% Female**	**% DMT (% escalation)**
Total brain volume	Total brain atrophy at y11	6	NA	−3.2 (−5.1 to −2.2)	29.3 (8.0)	83.3	83.3 (50.0)
	No total brain atrophy at y11	40	NA	−0.5 (−2.0 to 0.8)	33.8 (8.5)	75.0	47.5 (10.0)
	*p*-value	.	NA	*p* < 0.001	*p* = 0.22	*p* = 0.66	*p* = 0.10 (*p* = 0.03)
**Outcome measure iv**		***N***	**Mean thalamic volume Z-score compared to healthy controls at baseline**	**Mean thalamic volume Z-score compared to healthy controls at y 11 (range)**	**Mean age at baseline (SD) in years**	**% Female**	**% DMT (% escalation)**
Thalamic volume	Thalamic atrophy at y 11	21	NA	−3.6 (−8.0 to −2.0)	31.8 (8.6)	76.2	71.4 (23.8)
	No thalamic atrophy at y 11	25	NA	−0.9 (−2.0 to 1.0)	34.5 (8.3)	76.0	36.0 (8.0)
	*p*-value	.	NA	*p* < 0.001	*p* = 0.97	*p* = 0.99	*p* = 0.02 (*p* = 0.05)
**Outcome measure v**		***N***	**Mean T2 volume at baseline**	**Mean T2 volume at y 11 in mm^3^ (range)**	**Mean age at baseline (SD) in years**	**% Female**	**% DMT (% escalation)**
White matter lesion volume		46	NA	13626.2 (3525.3 to 51882.7)	33.2 (8.5)	76.1	52.2 (15.2)

*Cognition scores and MRI parameters were available for n = 49 and n = 46 individuals respectively; p-values are provided for the comparison of each outcome parameter between outcome group. HC, healthy controls; EDSS, extended disability status scores; SD, standard deviation; DMT, disease modifying therapy; NA, not available; n, number; y11, year 11 follow-up; % escalation indicates individuals which received second line DMTs.*

## Discussion

We interrogated the plasma proteome at diagnosis in 67 well characterized RRMS patients, and found no signature associated with dichotomized clinical, cognitive or MRI outcomes, or with white matter lesion volume 11 years after diagnosis. We used an innovative proteomics technology which allowed us to measure a predefined panel of 1310 proteins in plasma, including low abundant proteins. A subset of the proteins of the SOMAscan are of known relevance to MS, such as brain-derived neurotrophic factor, Chitinase-3-like protein 1 (aka YKL-40), hepatocyte growth factor, Contactin-2, and osteopontin (Teunissen et al., 2015). In addition, the panel includes proteins involved in inflammation and immune responses, and in CNS repair. Our cohort is representative of the demographics of MS in the general population, with female overrepresentation and mean age at disease onset in the early thirties. The proportion of individuals with whole brain atrophy was low, because we used a stringent definition of atrophy in order to retain high specificity. The proportion of DMT-treated individuals did not differ between outcome groups for the EDSS and cognitive function outcome measures, but was higher in individuals with thalamic atrophy compared to the group without. This finding may reflect the higher incentive to treat individuals with paraclinical signs for a more severe disease course, such as early thalamic atrophy. Several limitations may explain our results. First, our cohort consisted of individuals with a relatively mild disease course, and the differences in outcome measures between the dichotomised groups, although significant, were modest. Although this cohort may reflect the disease course of a majority of RRMS patients, a cohort with more dramatic differences in outcomes, if available, may be better suited for the exploratory nature of the study. Second, the size of the cohort was relatively small, yet larger than most of the MS cohorts investigated by proteomics and reported in the literature so far (Almeras et al., 2004; Avasarala et al., 2005; Rithidech et al., 2009; Byström et al., 2014; Jiang et al., 2014; Yin et al., 2014). Third, 47.8% of patients were treated with DMTs, which may modulate disease course. However, the proportion of DMT-treated individuals did not differ between outcome groups for the EDSS, global cognitive scale and whole brain volume outcome measures, indicating DMTs may not be a main determinant of outcome, 11 years after diagnosis in this cohort for these measures. Forth, the panel of proteins measured in plasma may in fact not have prognostic potential. However, in contrast to unbiased proteomic tools, this platform allows to measure low abundant proteins, including signaling molecules and brain-derived proteins, which could have reflected disease activity. Fifth, plasma storage time was long (12 years), and although the samples were collected within a few months of each other, processed identically, and stored according to international state-of-the-art guidelines on biobanking, storage time may have affected protein levels. Few studies have investigated the effect of long term storage on the plasma proteome. A mass spectrometry study found no difference in the number and intensity of peaks between plasma samples collected 4 years apart (Mitchell et al., 2005). The effect of longer storage times on the plasma proteome has not been investigated so far, but as storage time was the same for all samples, we do not expect a large influence on the results of this study. Sixth, plasma may not be a source for the discovery of early prognostic biomarkers. Indeed, the influence of peripheral organs on the blood proteome is high, and in this respect, cerebrospinal fluid may be better suited for biomarker discovery. Finally, disease prognosis may be difficult to predict at time of first clinical signs of disease, as many factors, amongst which infectious diseases, vitamin D levels, diet and lifestyle, may modulate disease course.

## Author Contributions

CB designed the study, analyzed the results, and wrote the manuscript. AE contributed the cognitive and MRI outcome measures and critically reviewed the manuscript. WvW performed the statistical analysis and critically reviewed the manuscript. MK-S did the proteomics experiments. CL provided the EDSS outcome measures and critically reviewed the manuscript. MS contributed the cognitive and MRI outcome measures and critically reviewed the manuscript. JK provided the EDSS outcome measures and critically reviewed the manuscript. CT designed the study and critically reviewed the manuscript.

## Conflict of Interest Statement

The authors declare that the research was conducted in the absence of any commercial or financial relationships that could be construed as a potential conflict of interest.

## Supplementary Material

DATA SHEET S1 Somalogic results.

## References

[B1] Almeras L. Lefranc D. Drobecq H. de Seze J. Dubucquoi S. Vermersch P. (2004). New antigenic candidates in multiple sclerosis: identification by serological proteome analysis. *Proteomics* 4 2184–2194. 15221778 10.1002/pmic.200300732

[B2] Avasarala J. R. Wall M. R. Wolfe G. M. (2005). A distinctive molecular signature of multiple sclerosis derived from MALDI-TOF/MS and serum proteomic pattern analysis: detection of three biomarkers. *J. Mol. Neurosci. Hum. Press* 25 119–126. 15781972 10.1385/JMN:25:1:119

[B3] Byström S. Ayoglu B. Häggmark A. Mitsios N. Hong M. G. Drobin K. (2014). Affinity proteomic profiling of plasma, cerebrospinal fluid, and brain tissue within multiple sclerosis. *J. Proteome Res.* 13 4607–4619. 10.1021/pr500609e 25231264

[B4] Colomba P. Fontana S. Salemi G. Barranca M. Lo Sicco C. Mazzola M. A. (2014). Identification of biomarkers in cerebrospinal fluid and serum of multiple sclerosis patients by immunoproteomics approach. *Int. J. Mol. Sci.* 15 23269–23282. 10.3390/ijms151223269 25517032 PMC4284765

[B5] Eijlers A. J. C. Meijer K. A. van Geest Q. Geurts J. J. G. Schoonheim M. M. (2018). Determinants of cognitive impairment in patients with multiple sclerosis with and without atrophy. *Radiology* 288 544–551. 29786489 10.1148/radiol.2018172808

[B6] Eijlers A. J. C. Meijer K. A. Wassenaar T. M. Steenwijk M. D. Uitdehaag B. M. J. Barkhof F. (2017). Increased default-mode network centrality in cognitively impaired multiple sclerosis patients. *Neurology* 88 952–960. 10.1212/WNL.0000000000003689 28179464

[B7] Goeman J. J. Van de Geer S. De Kort F. van Houwellingen H. C. (2004). A global test for groups fo genes: testing association with a clinical outcome. *Bioinformatics* 20 93–99. 14693814 10.1093/bioinformatics/btg382

[B8] Gold L. Ayers D. Bertino J. Bock C. Bock A. Brody E. N. (2010). Aptamer-based multiplexed proteomic technology for biomarker discovery. *PLoS One* 5:e15004. 10.1371/journal.pone.0015004 21165148 PMC3000457

[B9] Jiang S. F. Lu Q. Y. Hu S. Chen Y. Liu X. L. Yang Y. (2014). Proteomics comparison of the sera from multiple sclerosis patients and neuromyelitis optica patients. *Genet. Mol. Res.* 13 9292–9299. 10.4238/2014.February.14.3 24615106

[B10] Legroux L. Arbour N. (2016). Multiple sclerosis and T lymphocytes: an entangled story. *J. Neuroimmune Pharmacol.* 10 528–546. 10.1007/s11481-015-9614-0 25946987 PMC5052065

[B11] Liu J. Yin L. Dong H. Xu E. Zhang L. Qiao Y. (2012). Decreased serum levels of nucleolin protein fragment, as analyzed by bead-based proteomic technology, in multiple sclerosis patients compared to controls. *J. Neuroimmunol.* 250 71–76. 10.1016/j.jneuroim.2012.05.002 22633274

[B12] McDonald W. Compston A. Edan G. Goodkin D. Hartung H. P. Lublin F. D. (2001). Recommended diagnostic criteria for multiple sclerosis: guidelines from the international panel on the diagnosis of multiple sclerosis. *Ann. Neurol.* 50 121–127. 11456302 10.1002/ana.1032

[B13] McFarland H. F. Martin R. (2007). Multiple sclerosis: a complicated picture of autoimmunity. *Nat. Immunol.* 8 913–919. 17712344 10.1038/ni1507

[B14] Mitchell B. L. Yasui Y. Li C. I. Fitzpatrick A. L. Lampe P. D. (2005). Impact of freeze-thaw cycles and storage time on plasma samples used in mass spectrometry based biomarker discovery projects. *Cancer Inform.* 1 98–104. 19305635 PMC2657648

[B15] Rithidech K. Honikel L. Milazzo M. Madigan D. Troxell R. Krupp L. (2009). Protein expression profiles in pediatric multiple sclerosis: potential biomarkers. *Mult. Scler. J.* 15 455–464. 10.1177/1352458508100047 19324981

[B16] Smith S. M. Jenkinson M. Woolrich M. W. Beckmann C. F. Behrens T. E. J. Johansen-Berg H. (2004). Advances in functional and structural MR image analysis and implementation as FSL. *Neuroimage* 23(Suppl. 1), S208–S219. 15501092 10.1016/j.neuroimage.2004.07.051

[B17] Steenwijk M. D. Pouwels P. J. W. Daams M. Van Dalen J. W. Caan M. W. A. Richard E. (2013). Accurate white matter lesion segmentation by k nearest neighbor classification with tissue type priors (kNN-TTPs). *Neuroimage Clin.* 3 462–469. 10.1016/j.nicl.2013.10.003 24273728 PMC3830067

[B18] Teunissen C. Koel-Simmelink M. Pham T. Knol J. Khalil M. Trentini A. (2011). Identification of biomarkers for diagnosis and progression of MS by MALDI-TOF mass spectrometry. *Mult. Scler. J.* 17 838–850. 10.1177/1352458511399614 21505015

[B19] Teunissen C. E. Malekzadeh A. Leurs C. Bridel C. Killestein J. (2015). Body fluid biomarkers for multiple sclerosis—the long road to clinical application. *Nat. Rev. Neurol.* 11 585–596. 10.1038/nrneurol.2015.173 26392381

[B20] Teunissen C. E. Petzold A. Bennett J. L. Berven F. S. Brundin L. Comabella M. (2009). A consensus protocol for the standardization of cerebrospinal fluid collection and biobanking. *Neurology* 73 1914–1922. 19949037 10.1212/WNL.0b013e3181c47cc2 PMC2839806

[B21] Yin L. Liu J. Dong H. Xu E. Qiao Y. Wang L. (2014). Autophagy-related gene16L2, a potential serum biomarker of multiple sclerosis evaluated by bead-based proteomic technology. *Neurosci. Lett.* 562 34–38. 10.1016/j.neulet.2013.12.070 24406150

